# Non-native terrestrial slugs from Sinaloa, Mexico: *Deroceraslaeve* (O. F. Müller, 1774) and *Sarasinulaplebeia* (P. Fischer, 1868) (Mollusca, Gastropoda)

**DOI:** 10.3897/BDJ.10.e87666

**Published:** 2022-08-04

**Authors:** Laura Regina Alvarez-Cerrillo, Beatriz Yáñez-Rivera, Victoria Araiza-Gómez

**Affiliations:** 1 Centro de Investigación en Alimentación y Desarrollo, A. C. (CIAD), Unidad Mazatlán en Acuicultura y Manejo Ambiental, Av. Sábalo-Cerritos s/n, Estero del Yugo, C.P. 82100, Mazatlan, Mexico Centro de Investigación en Alimentación y Desarrollo, A. C. (CIAD), Unidad Mazatlán en Acuicultura y Manejo Ambiental, Av. Sábalo-Cerritos s/n, Estero del Yugo, C.P. 82100 Mazatlan Mexico; 2 Instituto Politécnico Nacional, Escuela Nacional de Ciencias Biológicas, Departamento de Zoología, Prolongación de Carpio y Plan de Ayala s/n, Col. Casco de Santo Tomás, 11340, Mexico City, Mexico Instituto Politécnico Nacional, Escuela Nacional de Ciencias Biológicas, Departamento de Zoología, Prolongación de Carpio y Plan de Ayala s/n, Col. Casco de Santo Tomás, 11340 Mexico City Mexico

**Keywords:** introduced, pest slug, COI, Agriolimacidae, Veronicellidae

## Abstract

This is the first record of two non-native terrestrial slug species from Sinaloa, Mexico. *Deroceraslaeve* and *Sarasinulaplebeia* were collected between 2019 and 2022 in Concordia and Mazatlan Municipalities (north-western Mexico). The external morphology and anatomic features of the dissected specimens coincide with the descriptions of each species, whose identities were also confirmed by their partial COI sequences. The ample occurrence of *S.plebeia* suggests that this species has an established population, while *D.laeve* was found as isolated individuals, likely associated with plant nurseries.

## Introduction

Non-native slug species can be responsible for the displacement of native species, crop damage and habitat destruction. Several ways of introduction into new ecosystems have been hypothesised, for example, commercial trade of plants or passive transportation of eggs and juveniles or adults attached to birds (Aubry et al. 2006, Barbato et al. 2017). The most updated assessment of non-native slugs in Mexico was performed by Naranjo-García and Castillo-Rodríguez (2017), who reported thirteen species: *Arioncircumscriptus* G. Johnston, 1828; *A.intermedius* Normand, 1852; *Boettgerillapallens* Simroth, 1912; *Derocerasinvadens* Reise, Hutchinson, Schunack & Schlitt, 2011; *D.laeve* (O. F. Müller, 1774); *D.reticulatum* (O. F. Müller, 1774); *Ambigolimaxvalentianus* (A. Férussac, 1821) (listed as *Lehmanniavalentiana*); *Limacusflavus* (Linnaeus, 1758); *Limaxmaximus* Linnaeus, 1758; *Milaxgagates* (Draparnaud, 1801); *Phyllocaulisgayi* (P. Fischer, 1871) and *Sarasinulaplebeia* (P. Fischer, 1868). Recently, *Arionvulgaris* Moquin-Tandon, 1855 was recorded by Araiza-Gómez et al. (2021).

The non-native slugs previously reported in literature for the State of Sinaloa are *A.valentianus* (*Naranjo-García and Castillo-Rodríguez 2017*), *P.gayi* and *S.dubia* (Naranjo-García et al. 2007). However, in the same reports, the authors argued the need to confirm the occurrence of *P.gayi* due to the absence of records since 1925. The knowledge of native slugs from Sinaloa is limited. Citizen observations in iNaturalist (https://www.naturalista.mx/projects/moluscos-de-Mexico), suggest the presence of *Leidyulamoreleti* (Crosse & Fischer, 1872). The occurrence of the species was confirmed by us using the external morphological characteristics of the species given the limitations of the platform.

The native origin of *D.laeve* remains uncertain; its actual distribution seems to be cosmopolitan (Gittenberger et al. 2018). It has been hypothesised that the species is Palearctic in origin and subsequently spread throughout Europe (Wiktor 2000). However, several previous records suggest a Holarctic origin of the species (Sysoev and Schileyko 2009), capable of colonising a wide range of habitats due to its huge ecological plasticity. The genus *Deroceras* (Agriolimacidae) includes at least 123 species (Wiktor 2000). In Mexico, *D.laeve* was first reported from the State of Veracruz (south-eastern Mexico) (Strebel and Pfeffer 1880), with recent records from Chihuahua and Durango (northern Mexico) (Araiza-Gómez et al. 2017).

*Sarasinulaplebeia* was described from New Caledonia (Oceania). Darrigran et al. (2020) commented that its origin in South America is unknown. Thomé (1993) explains the presence of this species in Rio de Janeiro, Brazil concerning changes in taxonomy and nomenclature due to the native species *Vaginulabehni* Semper, 1885, which was later synonymised with *S.plebeia*. Recent reports as a non-native species were documented in North and Central America (Daglio et al. 2020). The first record of *S.plebeia* in Mexico was from Chiapas and Veracruz (Andrews and Dundee 1987), with most records occurring in the centre and south of the country and some records from the north of Mexico. In the current contribution, we present new distributional records of *D.laeve* and *S.plebeia* from Sinaloa. We support these records with anatomic and molecular data.

## Material and methods

Manual collection for living slugs were carried out between August 2019 and April 2022 in different urban and rural locations of six municipalities in the State of Sinaloa (north-western Mexico), but these species were only found in two: Concordia and Mazatlan (Table 1). In total, 122 specimens of *Sarasinulaplebeia* and three of *Deroceraslaeve* were collected.

The specimens were relaxed in a jar with water until fully stretched and died (~ 12 h). Some specimens were photographed alive using a digital camera (Lumix DMC–FS3, Panasonic). Once dead, the cleaning of mucus was performed in a sieve under running cold water. The slugs were then fixed on 90% ethanol.

All specimens were examined for external morphology and 25 slugs of *S.plebeia* and two of *D.laeve* were dissected. Two specimens of *S.plebeia* and one of *D.laeve* (which was damaged during processing), were selected for molecular analysis.

DNA extraction of the tissue of the foot muscle was performed using the Blood and Tissue kit according to the manufacturer's specifications (QIAGEN, California, USA). The integrity and quality of the DNA was verified on an agarose gel by electrophoresis. The COI gene was amplified by PCR using COIF and COIR (López et al. 2019). The reaction mixture consisted of 50 ng of DNA, 0.6 μl of each 10 mM primer, 1.5 μl of 10x buffer, 0.6 μl of 10 mM dNTPs, 1.5 μl of 50 mM MgCl_2_ and 0.2 μl of Taq Polymerase (Invitrogen) in a total volume of 25 µl. The amplification conditions were initial denaturation at 94°C for 5 min, followed by 35 cycles at 94°C for 30 s, 50°C for 30 s, 72°C for 30 s and a final extension at 72°C for 7 min.

The PCR products were purified with the GFX™ PCR DNA and GelBand Purification Kit (GE Healthcare, Buckinghamshire, UK), then sequenced by Macrogen Inc., Korea. The nucleotide sequences were compared with the sequences deposited in the gene bank (GenBank) of the National Center for Biotechnology Information (http://www.ncbi.nlm.nih.gov), with the BLASTn algorithm. The sequences obtained were deposited in GenBank (Access number: ON678123–25).

A taxonomic assignment was carried out using a phylogenetic inference analysis by Maximum Likelihood (ML) with the algorithm implemented in PhyML v. 3.0 (Guindon et al. 2010). The nucleotide substitution model that best fits the data was determined with SMS: Smart Model Selection in PhyML (Lefort et al. 2017). The evolutionary selected model by the Akaike Information Criterion for each dataset (Suppl. material 1) was the HKY85 model with gamma distribution, shape = 0.173 for *Deroceras* and shape = 0.566 for *Sarasinula*. To estimate the reliability of each node, a bootstrap procedure was performed with 1000 pseudoreplicates. We employed as outgroup *Limaxmaximus* (KF894386, KM612139) for the genus *Deroceras* and *Onchidium* (MN528062, KX179520) for the genus *Sarasinula*.

## Results

**Family Agriolimacidae**

**Figure 1.**
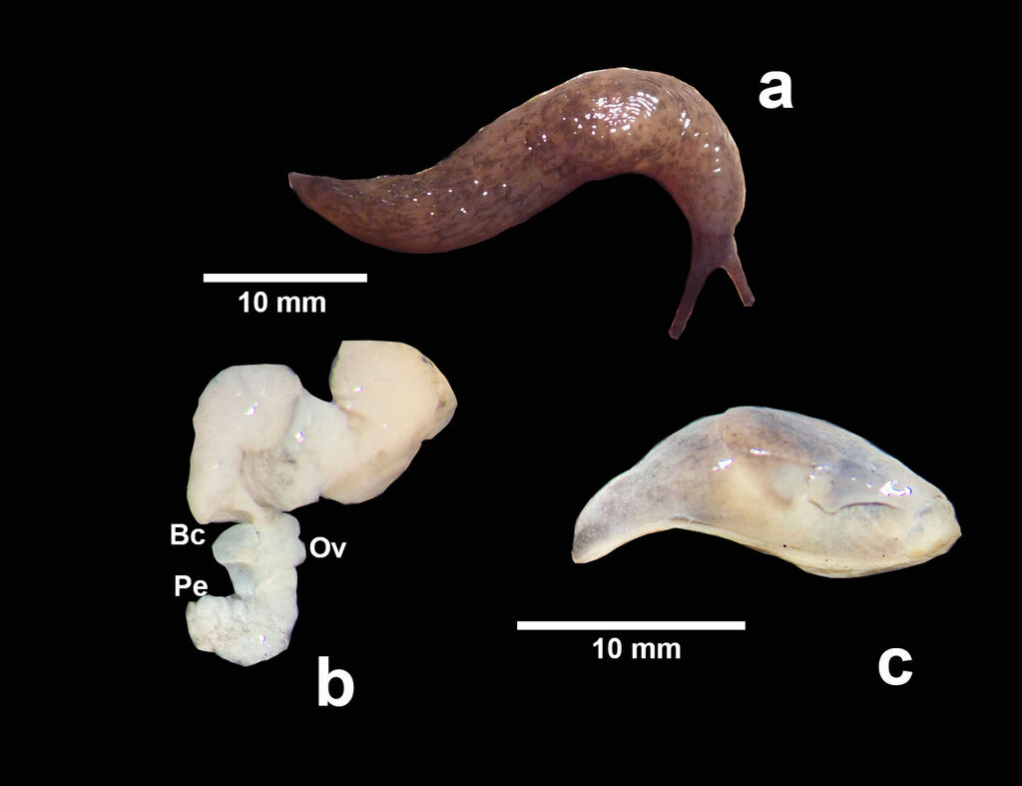
*Deroceraslaeve*: **a** live specimen; **b** genitalia (Bc: bursa copulatrix, Ov: oviduct, Pe: penis); **c** external appearance of a preserved specimen.

***Deroceraslaeve* (O. F. Müller, 1774)** (Fig. 1)

**Morphology**: Live pigmentation grey and dark brown (Fig. 1a), ocular tentacles almost black, with concentric striations in the mantle characteristic for the genus. Dorsum with tubercles, distally with barely noticeable keel. Body length of preserved specimens, 18-22 mm. The bursa copulatrix on the oviduct and a barely visible penis were observed (Fig. 1b). The aphallic or phallic form was unconfirmed, possibly related to the size of the specimen and preservation state.

**Figure 2.**
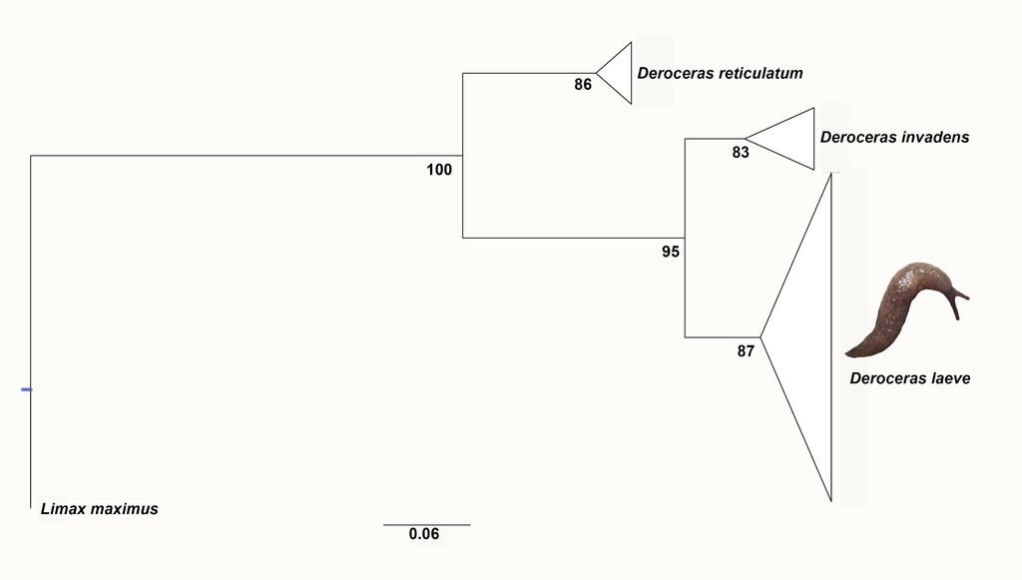
Maximum Likelihood (ML) phylogenetic tree reconstruction of *Deroceras* using 310 bp of the COI. Numbers on branches indicate ML bootstrap values. GenBank access numbers of sequences employed in supplementary material (Suppl. material 1).

**Molecular markers**: A fragment of 717 bp was obtained. After editing, a fragment of 661 bp was used for the BLAST analysis and another of 310 bp for the phylogenetic analysis. The sequence obtained in this study had 98.22% nucleotide identity of similarity with sequence KX959495.1 registered in GenBank as *D.laeve*. The sequence clustered closer in the phylogeny with specimens from Mexico (KX959494, KX959501) and Canada (MG421943) (Fig. 2).

**Remarks**: The main differences between *D.laeve* and other species of the genus are related to the mantle coverage, pigmentation and reproductive features (Araiza-Gómez et al. 2017). *Deroceraslaeve* and *D.invadens* share the mantle length that covers almost half the body and have similar sizes (~ 24 mm and ~ 28 mm, respectively). However, these two species differ in pigmentation; *D.invadens* has a pale greyish body with a few spots on the creamy-brown mantle. The differences with *D.reticulatum* are the cover of the mantle that reaches one third of the body length (~ 35 mm) and pale brown pigmentation, almost white with dark spots, with the sole cream-coloured. The pigmentation patterns in *D.laeve* include light brown, grey to almost black, some with a speckled mantle, the sole cream-coloured and others black delineated (Araiza-Gómez et al. 2017).

Regarding reproductive features, *D.laeve* can be found in two forms: the phallic form with a long slender penis and the aphallic form with greatly reduced or missing male reproductive organs (Wiktor 2000, Araiza-Gómez et al. 2017). The variant of this species was unconfirmed due to the preservation condition. *Derocerasreticulatum* has a penial gland on the proximal part of its penis with a flagellum having a variable number of bulbous branches. *Derocerasinvadens* shows two side pockets, the penial lobe and caecum of roughly equal width, but with a longer penial lobe, both having rounded tips; gland fingers long mid-way between the pockets and the retractor muscle attaches between the lobe and caecum.

**Habitat**: The three specimens were collected in gardens and from plants recently purchased at plant nurseries. The first individual was collected from *Spathiphyllum* spp. in an urban house. The other two were collected from *Euphorbiapulcherrima* in an agricultural/rural area. Globally, *D.laeve* inhabits an extremely wide range of habitats (Dedov et al. 2020).

**Family Veronicellidae**

**Figure 3.**
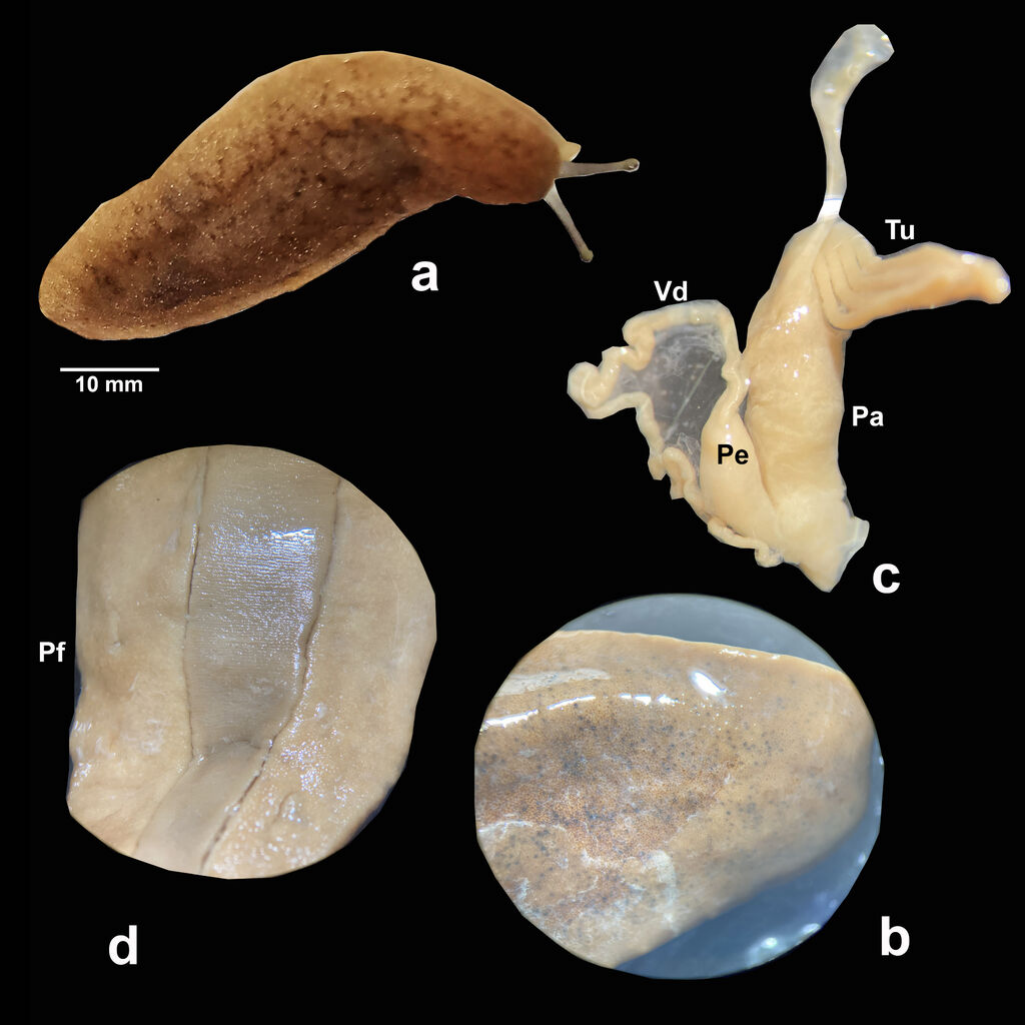
*Sarasinulaplebeia*: **a** live specimen; **b** punctuations on the notum of the specimen; **c** male reproductive system (Pa: papilla of the digitiform gland, Pe: penis, Tu: digitiform tubules, Vd: vas deferens); **d** hyponotum (Pf: female genital pore).

***Sarasinulaplebeia* (P. Fischer, 1868)** (Fig. 3)

**Morphology**: Live pigmentation brown (Fig. 3a), with scattered small punctuations in the thickened notum (Fig. 3b); light grey after preservation. Body length 45.26 ± 10.92 mm (min = 20.75 mm, max = 57.95 mm, n = 122). Penis short, smooth, without annular protrusion, bilaterally symmetrical, with enlarged glands, even the digitiform gland (Fig. 3c) with an elongated form; with four to six tubules subequal in length, but some individuals showed one shorter than the rest.

**Figure 4.**
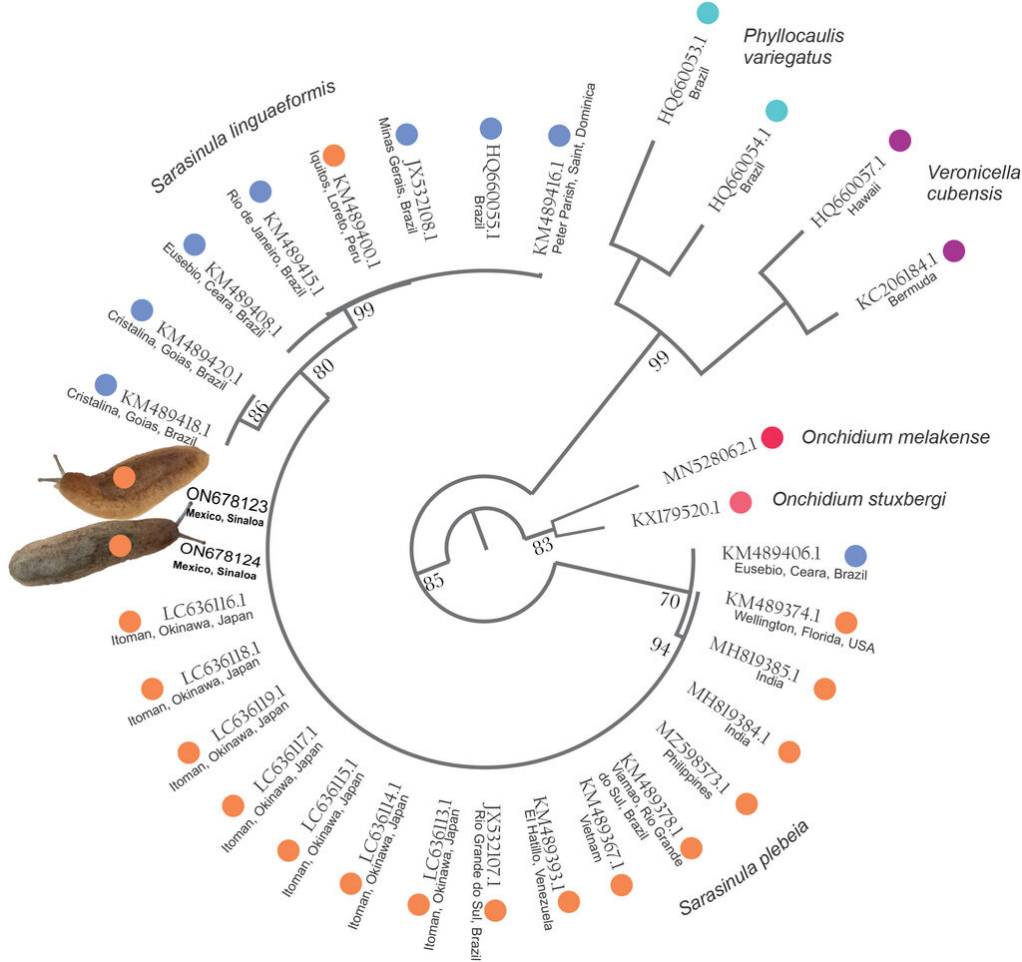
Maximum Likelihood (ML) phylogenetic tree reconstruction of *Sarasinula* using 470 bp of the COI. Numbers on branches indicate ML bootstrap values. Tip label is the accession number of GenBank and the country of origin.

**Molecular markers**: A fragment of 800 bp was obtained. After editing, a fragment of 621 bp was used for the BLAST analysis and another of 470 bp for phylogenetic analysis, matching the length of the sequences in GenBank. The sequences (n = 2) had 100% of similarity with sequence JX532107.1 registered in GenBank and 99.83% with sequences MZ598573.1, KM489367.1, both identified as *S.plebeia*. Mexican sequences clustered closer in the phylogeny with specimens from Okinawa, Japan (Fig. 4).

**Remarks**: The morphology of the penis is the main feature to differentiate *S.plebeia* and *S.dubia*. The penis is club-shaped in *S.plebeia* and tapering distally in *S.dubia*; identification of veronicellid slugs is valid when characters of sexual anatomy and penial gland are taken into consideration (Thomé 1989). We observed a slight variation in digitiform tubules (four to six).

**Habitat**: Abundant in gardens of urban houses. Only three specimens were found in natural vegetation next to a tributary of the Panuco River (near an abandoned mine). Globally appears limited to tropical environments.

**Figure 5.**
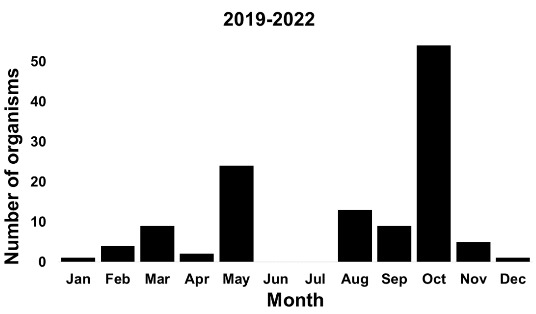
Temporal distribution of *S.plebeia* during the sampling along the years 2019–2022 (n = 122).

Specimens of *S.plebeia* were collected in all months, except for June and July between 2019 and 2022 (Fig. 5). In these months, the mean temperature increases with relatively less humidity than in August, the hottest month in summer. This probably limits the activity of slugs.

## Discussion

The list of species of non-native terrestrial slugs from Mexico, provided by Naranjo-García and Castillo-Rodríguez (2017), already includes both species reported herein. However, their data include limited coverage of localities in northern Mexico. The occurrence of *Deroceraslaeve* and *Sarasinulaplebeia* in the State of Sinaloa are the first records for this region. The introduction of terrestrial gastropods is related to horticulture, agriculture and ornamental plants (parks and gardens), in at least 20% of the cases. The vectors of the 40% of documented introductions are unknown (Darrigran et al. 2020). Our findings indicate that both species could have been introduced in this area via nursery plants, occurring mostly in gardens, but the presence of *S.plebeia* in natural vegetation suggests that this species is already invading natural habitats.

Impacts related to biological invasions have a direct effect on biodiversity loss (Bellard et al. 2021, Dueñas et al. 2021). In addition, introducing invasive slugs, mainly the *Sarasinula* species, is related to dry-bean and maize crop damage in Central America (Rueda et al. 2002). Although it appears that their effect as agricultural pests has been reduced by improving management by farmers, it remains as a vector for parasites for rodents and other mammals and humans (Nurinsiyah and Hausdorf 2018). Spatio-temporal occurrence of *S.plebeia* indicates an actual expansion process and their genetic convergence with Japanese and South American individuals demonstrate their invasive potential (Hirano et al. 2022).

The widespread distribution of both species could be true. In Pakistan, Havlác (2004) discussed the role of gardening activities in the population establishment of *D.laeve*. This species is the most widely distributed in the country, although the spread of three species of the genus (*D.laeve*, *D.invadens*, *D.reticulatum*) is a fact in Mexico (Araiza-Gómez et al. 2017). *Deroceraslaeve* still have a limited distribution in the region and is restricted to winter conditions and in plant species commonly used in gardening. This species has been registered as a pest in crops, such as cabbages, maize, soybean, amongst others, being a pest in agriculture and horticulture worldwide (Byers and Calvin 1994, Gittenberger et al. 2018). The genetic convergence of individuals from Sinaloa is related to other Mexican localities, such as Mexico City and the States of Oaxaca, Puebla, Queretaro and San Luis Potosi (GenBank access: KX959492-99, KX959500-01) and from Canada.

The establishment of non-native species requires favourable local conditions and temperature and humidity seem to be crucial, for the development of the population and further expansion into surrounding natural habitats (Dedov et al. 2020). The reproductive characteristics of non-native species play a crucial role in their invasive potential. Both species here considered are hermaphrodites with a self-fertilisation strategy (Rueda et al. 2002, Clemente et al. 2007). The development of *D.laeve* has two phases, a juvenile stage of pre-oviposition and a mature stage of oviposition during which the slugs lay their eggs; self-fertilisation is its normal breeding system; isolated individuals can produce fertile eggs. The reproduction occurs either in autumn or spring, once during their lifetime (Faberi et al. 2006). The individuals of *S.plebeia* can function as both male and female during their lifetime and self-fertilisation may occur in isolation (Rueda et al. 2002). The reproductive maturity occurs at ~ 2.5 months of age. An organism can produce one to four clutches per year with approximately 30 eggs. Reproduction is generally high during the rainy season (Naranjo-García et al. 2007).

Biodiversity inventories require reliable species identification, but in terms of biological invasions, the correct species assignment is essential. In this regard, the high number of synonyms is related to the use of few morphological characters for species delimitation or superficial revision, based on external features and pigmentation patterns (Maceira F. 2003). The taxonomy of the slugs requires analysing their internal anatomy of male genitalia and the use of molecular markers (Hirano et al. 2022), a crucial requirement to understand the dispersion of invasive species. For instance, considering that *S.plebeia* is one of the two species with the widest distribution (Gomes and Thomé 2004), further studies on this species could use molecular data to identify clades associated with introduction routes and spreading.

The main contribution of this paper consists of new records of two non-native species supported by molecular data, as a step towards the better understanding on terrestrial slugs invasions.

## Supplementary Material

30300014-BFD3-5AC4-A18C-F5A1E0B4924F 10.3897/BDJ.10.e87666.suppl1 Supplementary material 1 GenBank accession numbers of sequences used in the phylogenetic inference of *Deroceraslaeve* (Fig. 2)
Data type GenBank accession numbers of sequences, localities Brief description In this table the species, number of sequences of GenBank and localities of the specimens are listed. In total were used 143 sequences: 20 for *Derocerasinvadens*, 101 for *D.laeve*, 20 for *D.reticulatum* and two for outgroup *Limaxmaximus*. File: oo_716731.csv https://binary.pensoft.net/file/716731 Araiza-Gómez Victoria, Alvarez-Cerrillo Laura Regina, Yáñez-Rivera Beatriz

## Figures and Tables

**Table 1.** Table 1. Slugs collected in two municipalities of Sinaloa State, Mexico. Urban (*), agricultural/rural (**).

Species	Municipality	Locality	Altitude (m a.s.l.)	Coordinates	n
***Deroceraslaeve***	Mazatlan	*Portomolino	11	23.26263, -106.406	1
		**Siqueros	28	23.339309, -106.239243	2
***Sarasinulaplebeia***	Mazatlan	*Marivento	11	23.278083, -106.429989	112
		*Cerro del Vigia	38	23.190944, -106.425833	7
	Concordia	**Panuco	656	23.428808, -105.896592	3
